# Four decades of under-representation: a qualitative study to gain young adult insights to transform inclusive recruitment in clinical research

**DOI:** 10.1136/bmjopen-2026-122025

**Published:** 2026-10-26

**Authors:** Kanta Kumar, Vanshika Agarwal, Palak J Trivedi, Sheila Greenfield, Elaine YL Leung, Lorraine Jacques, Jonathan I Quinlan

**Affiliations:** 1 College of Medicine and Health, School of Health Sciences, University of Birmingham, Birmingham, UK; 2 Department of Rheumatology, Royal Wolverhampton NHS Trust, Wolverhampton, UK; 3 School of Sport, Exercise and Rehabilitation Sciences, University of Birmingham, Birmingham, UK; 4 Liver Unit, University Hospitals Birmingham, Birmingham, UK; 5 National Institute for Health and Care Research (NIHR) Birmingham, Centre for Liver and Gastrointestinal Research, School of Infection, Inflammation and Immunology, University of Birmingham, Birmingham, UK; 6 Department of Applied Health Research, University of Birmingham, Birmingham, UK; 7 Department of Cancer and Genomic Sciences, University of Birmingham, Birmingham, UK; 8 Royal Wolverhampton NHS Trust, Wolverhampton, UK; 9 National Institute for Health and Care Research (NIHR) Biomedical Research Centre: Birmingham, Birmingham, UK

**Keywords:** Research Design, QUALITATIVE RESEARCH, PUBLIC HEALTH, Young Adult, Health Equity

## Abstract

**Abstract:**

**Background:**

Over the past four decades, ethnic minority groups have remained persistently under-represented in UK health research, despite longstanding evidence of recruitment inequities. This under-representation limits research relevance and impacts clinical outcomes, particularly for communities disproportionately affected by chronic conditions. Young adults may offer an important route to engaging families and wider communities, yet their perspectives on research participation remain underexplored.

**Objectives:**

This qualitative study aimed to (1) explore the views of young South Asian and Black adults on taking part in health research themselves; and (2) understand how they believe their families and wider communities could be effectively approached and supported to participate

**Setting and design:**

Semi-structured interviews were conducted remotely with young adults residing across England. Aggregated recruitment data were obtained from a West Midlands National Health Service (NHS) Trust to examine patterns of research participation in research from different racial and minority ethnic backgrounds and provide local context of our interview findings. The results have been reported using the Consolidated Criteria for Reporting Qualitative Research guidelines.

**Participants:**

22 young adults (19–30 years) who self-identified as Southeast Asian (n=20) or Black (n=2) took part in the study. Trust data covered 524 participants recruited into commercial and non-commercial research studies in 2025.

**Results:**

NHS Trust data revealed marked under-representation of ethnic minority groups: 76.5% of participants were White with only 11.6% Asian and 4.8% Black participants in non-commercial studies (compared with local population of 60.5% White, 21.1% Asian and 9.3% Black), with no Black participants recruited into commercial studies. Regarding the interviews, four themes were evident: (1) generational and cultural influences on health practices; (2) experiences and barriers in health research participation; (3) motivators including modest incentives, non-invasive designs and peer endorsement; and (4) communication and trust-building.

**Conclusions:**

Young adults may represent an underutilised bridge between researchers and ethnically diverse families and communities. Improving participation requires culturally attuned and transparent approaches, trusted communication and sustained organisational commitment to inclusion. The findings identify practical strategies for supporting more inclusive recruitment and have informed the development of a short, evidence-informed video resource for researchers (https://tinyurl.com/4z5mudp3).

**STRENGTHS AND LIMITATIONS OF THIS STUDY**

- The interview guide was constructed with direct input from patient and public involvement and refined through public partners of South Asian and Black heritage.
- Our findings build on existing literature through the inclusion of young adults that are often overlooked in health-related research.
- Despite our efforts, Black adults were under-represented, likely reflecting the demographics of our existing recruitment networks, which limited the diversity of perspectives and comparative insights across ethnic groups.

## Introduction

 Health research shapes clinical practice, guides public health action and drives improvements in care.^1^ Yet for more than 30 years, researchers have consistently documented the under-representation of South Asian and Black communities in UK health studies, including clinical trials, observational cohorts and interventional research.^2 3^ These communities are ever-expanding within the UK and currently comprise approximately 13% of the UK population (7.8 million people). Therefore, this persistent omission limits who benefits from research, whose experiences shape medical knowledge and whose health outcomes improve as a result. The issue is not abstract: ethnic minority communities experience a disproportionate burden of long-term conditions such as type 2 diabetes, hypertension, cardiovascular disease and inflammatory disorders, often presenting earlier than White populations.^4–7^ When the very groups most affected by disease remain absent from research, inequalities deepen.^8^

The COVID-19 pandemic brought these inequities into sharp public focus; patients in ethnic minority groups were disproportionately linked to higher morbidity and mortality even after socioeconomic confounders are considered.^8 9^ The physiological basis of health outcomes in ethnically diverse populations is under-explored. Communities now openly question why research, clinical trials and health interventions continued to overlook them.^10 11^ Public awareness grew, media attention intensified and communities demanded that research finally reflect their lived realities.^12 13^ Despite national efforts to diversify recruitment during COVID-19 vaccine and therapeutic trials, systematic reviews showed persistent under-representation of Black, Asian and mixed ethnic groups, even when trials were explicitly designed to be inclusive.^14^ In many ways, the pandemic exposed what decades of research had already indicated: deep-rooted structural barriers and mistrust still limit who is invited, who participates and who benefits from research.^15 16^ These issues are especially visible in regions such as the West Midlands, one of the most ethnically diverse areas in England.^17^

Young adults often act as interpreters, advocates and cultural mediators for older relatives who may face language barriers, limited digital literacy or unfamiliarity with research processes.^18^ They are simultaneously embedded in traditional community networks and exposed to contemporary health information through education, peers and digital platforms.^19 20^ This dual positioning gives young adults unique insight into how research is perceived within families, how trust is built or lost and how participation decisions are negotiated across generations. As such, young adults occupy a critical but often overlooked role in this landscape.

Guided by this context, our study pursued four interconnected aims.

1. To quantitatively evaluate National Health Service (NHS) trial recruitment data within the UK West Midlands.
2. To understand how cultural traditions and values influence approaches in making health-related decisions.
3. To explore how young South Asian and Black adults view their own participation in health research and whether this aligns with long-standing patterns of exclusion.
4. To understand how these young adults believe their parents, elders and community members could be effectively engaged in research, effectively positioning them as potential ambassadors for inclusive approaches.

## Methods

### Study design

We conducted an in-depth qualitative study with 22 young adults (19–30 years) to explore how young adults from South Asian and Black communities understand, negotiate and engage with health research, including their views on how best to engage older community members.

This study was grounded in an interpretivist paradigm, which recognises that individuals construct meanings through their social, cultural and lived experiences, rather than assuming a single objective reality.^21^ This approach was appropriate because our aim was to understand how young adults from South Asian and Black communities perceive and make sense of health research participation within their family, cultural and intergenerational contexts. Methodologically, this informed an inductive and reflexive approach to data collection and analysis, enabling participants’ accounts to be interpreted within the social and cultural contexts in which they were produced.^21^ Researchers also maintained reflexive awareness of how their own cultural and professional positioning could influence data collection and interpretation.

We conducted semi-structured interviews to enable participants to speak freely while allowing researchers to probe salient issues in greater depth. We report the study in accordance with the Consolidated Criteria for Reporting Qualitative Research (COREQ) to ensure transparent reporting of reflexivity, study design, conduct and analysis.

### NHS study recruitment data

To contextualise the qualitative findings and to quantify existing recruitment disparities within our local region, we sought to obtain anonymised recruitment data from the Royal Wolverhampton NHS Trust Research and Development Department. No identifiable information was shared; only aggregated data on ethnicity, study enrolment, the project type and specialty were provided. Data provided included enrolled study participants within the NHS Trust as of January 2025.

### Participant recruitment and sampling

Eligibility criteria included being aged 18–30 years, residing in the UK and self-identifying as South Asian or Black. Recruitment aimed for maximum variation sampling to reflect diversity across gender, educational attainment and socioeconomic background, although the final sample was weighted towards South Asian adults.

Recruitment strategies were primarily community-based, reflecting the study’s focus on populations that are often under-represented in health research. Participants were approached through informal and peer-led channels such as WhatsApp groups, word-of-mouth and community networks, as well as through outreach via student societies and local cultural groups. These informal networks, relationships and links have been previously established by members of the research team. Snowball sampling was also employed, with existing participants recommending peers who met the eligibility criteria. Previous research participants were only contacted where written informed consent had been previously provided. This approach was utilised to help build trust and ensured accessibility among individuals who might not typically engage with institutional or clinical research invitations. All participants were offered a voucher with a £50 value as remuneration for their time.

In total, 33 individuals contacted the research team of whom 25 responded to follow-up and expressed interest to join the study. Following eligibility screening, 22 participants were enrolled and completed individual interviews. Reasons for declining participation included limited availability, uncertainty about the relevance of the study to their own experiences and general disinterest. Recruitment continued until thematic sufficiency was achieved^22^ (ie, repetition of opinion and responses was apparent, and no new substantial insights emerged in the final interviews). Participant demographics are presented in table 1, and the recruitment process is illustrated in figure 1.

**Figure 1:**
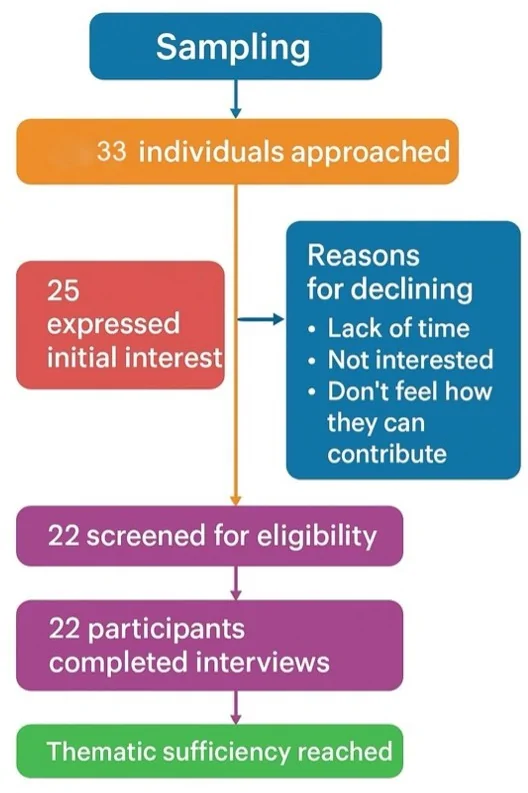
Recruitment flow for the Highlight study: 33 individuals were approached, of whom 25 expressed interest and were screened for eligibility. 22 young adults met inclusion criteria, provided consent and completed interviews. Recruitment was concluded once thematic sufficiency was reached.

**Table 1:** Ethnicity data collected from The Royal Wolverhampton NHS Trust for enrolled study participants as of January 2025

Ethnicity category	N (%)
White—British	387 (73.8)
Asian or Asian British—Indian	50 (9.5)
Not stated	25 (4.7)
Black or Black British—Caribbean	13 (2.4)
Black or Black British—African	10 (1.9)
White—any other White background	7 (1.3)
Not known	7 (1.3)
Asian or Asian British—Pakistani	5 (0.9)
Asian or Asian British—any other Asian background	5 (0.9)
Other ethnic groups—any other ethnic group	3 (0.6)
Mixed—White and Black Caribbean	3 (0.6)
Mixed—White and Black African	3 (0.6)
Black or Black British—any other Black background	2 (0.4)
Mixed—any other mixed background	2 (0.4)
Asian or Asian British—Bangladeshi	1
Mixed—White and Asian	1

NHS, National Health Service.

### Development of the interview topic guide

The interview guide (online supplemental material 1) was systematically developed in alignment with COREQ criteria, specifically addressing Domain 1 (Research Team and Reflexivity) and Domain 2 (Study Design), which emphasise comprehensive reporting of development, piloting and interviewer characteristics. The interview guide was provisionally designed by the research team in order to address the aforementioned aims and research questions. The interview guide was then shared with public partners of the relevant population and followed an iterative refinement process to ensure all possible angles could be covered.

#### Literature mapping and content identification

We conducted a targeted scoping review of existing studies on youth and young adult engagement in health research. Specifically, we focused on both general research participation and single-disease studies (eg, diabetes, nutrition or other condition-specific research) to identify common motivators, barriers, and ethical considerations. Key themes extracted included awareness of research, motivations for participation, barriers (practical, cultural, intergenerational), incentives, communication preferences and involvement of family members.

#### COR**EQ-informed structuring**

Items were organised to ensure comprehensive coverage of COREQ-recommended elements, including (1) awareness and understanding of research; (2) motivations and barriers, including social, cultural and intergenerational factors; (3) perceptions of research burden and incentives; (4) preferences for recruitment and communication channels and (5) strategies to engage older relatives or gatekeepers. For each topic, we included open-ended questions, followed by probes, consistent with COREQ recommendations to allow depth while maintaining comparability.

#### Piloting and iterative refinement

We piloted the guide within the team and made minor iterative adjustments to improve clarity and cultural relevance before piloting with mock interviews conducted by VA and KK with two volunteers relevant to the target population. Feedback focused on clarity, cultural sensitivity, flow and accessibility. Minor iterative revisions were made to improve comprehensibility and cultural appropriateness. Data collected from the mock interviews was not utilised in the final analysis.

#### Interviewer training

VA (Indian female public health researcher) and KK (British Indian female, associate professor, rheumatology) underwent additional training on culturally sensitive, reflexive qualitative interviewing, including role-play, peer feedback and review of pilot interviews. Both maintained reflexive awareness of positionality, potential biases and strategies to reduce social desirability bias.^23^

#### Documentation of development

Detailed records were kept of each version of the guide, pilot feedback and final revisions to ensure replicability. Reflexive memos captured decisions on question order, probing strategy and inclusion/exclusion of items.

### Data collection

Interviews were conducted remotely via secure video-conferencing platforms between February and March 2025. Each interview lasted 45–60 min and was conducted by trained researchers (VA and KK) experienced in intercultural qualitative interviewing. Field notes captured contextual features (tone, hesitations, affect) to support interpretive depth.

### Data analysis and thematic analysis

#### Familiarisation

VA, KK and JIQ (White male, assistant professor in exercise physiology) independently read all transcripts multiple times to immerse themselves in the data and note initial impressions, recurring patterns and contextual nuances, including tone, hesitations and culturally specific expressions. Reflexive notes supported interpretive depth.

#### Coding framework

Inductive coding was performed manually by VA, KK and JIQ using printed transcripts and structured coding tables. Both semantic (explicit) and latent (underlying) meanings were captured, and following independent coding, the three researchers met to compare and agree on all codes, resolving discrepancies by consensus. Agreed codes were collated into a provisional framework using shared coding tables, including code definitions and illustrative quotes. This framework was then discussed with the wider team (SG, PT, EL) for review and refinement. Any decisions regarding merging, splitting or redefining codes were documented in reflexive memos.

### Theme identification, refinement and defining themes

Following the agreement of the code framework, codes were consequently grouped into potential themes following Braun and Clarke’s (2006) six-phase approach. Specifically, VA, KK and JIQ iteratively revisited transcripts to ensure themes truly represented participants’ perspectives. Agreed themes and sub-themes were reviewed and validated by the wider team (SG, PJT, EL), ensuring interpretive credibility and minimising individual bias. Themes were then given clear, descriptive names and sub-themes where necessary. Illustrative verbatim quotes, with participant identifiers and demographic details, were selected to support transparency.

### Ensuring rigour

Rigour was enhanced through several complementary approaches. Triangulation strengthened the credibility of our interpretations by reducing individual researcher bias. Herein, three researchers independently coded transcripts, with any discrepancies resolved through achieving consensus and themes were subsequently reviewed by the wider team and unanimously agreed on. Reflexivity enabled critical consideration of researcher influence, with positionality, assumptions, and analytical decisions documented in reflexive memos. In addition, an audit trail was utilised to enhance transparency by retaining coding tables, memos, and records of theme development. Analysis continued until no substantively new themes or insights emerged in order to support thematic sufficiency. Finally, member checking enhanced credibility by sharing our finding summaries with two study participants to confirm that interpretations accurately reflected their perspectives.

### Researcher reflexivity and positionality

VA and KK conducted all interviews, and all three coders (VA, KK, JIQ) reflected on their professional and personal backgrounds, including cultural familiarity and potential influences on participant engagement and interpretation. Reflexive discussions informed coding, theme development, and interpretation, in line with COREQ recommendations.

### Patient and public involvement statement

Patients and public were involved in the planning of the study through their involvement in designing the interview guide as noted above.

## Results

### Local recruitment context

A total of 524 participants were enrolled in research studies at the Trust as of January 2025. Within this data set, White participants comprised 76.5%, followed by South Asian participants (11.6%), Black participants (4.8%), mixed race (0.4%), other (0.6%) and not stated/unknown (6.1%). The majority of participants were recruited to non-commercial compared with commercial studies (91.8% vs 8.2%). Further breakdown by study specialty revealed that under-representation was most pronounced in oncology studies, followed by cardiovascular and neurology research. In contrast, slightly higher participation from Asian groups was seen in diabetes- and metabolic-related studies, reflecting the higher disease burden in these populations. Notably, no Black participants were recorded in commercial projects data can be seen in table 1.

### Interview participant demographics

A total of 22 young adults from South Asian (n=20) and Black (n=2) backgrounds took part in individual semi-structured interviews. The majority were female (n=17), with ages ranging from 19 to 30 years (mean 26.8±3.3 years). Participants varied in educational background, with many having a university degree or postgraduate study. These characteristics provided a diverse set of perspectives while still reflecting the lived realities of younger ethnic minority adults in the UK. Full demographic data of interview participants can be seen in table 2.

**Table 2:** Demographic data for all participants included in the interview component of this study (n=22*)*

Characteristics	n=22
Age	26.8±3.3 years
Sex (m/f)	(5/17)
Country of birth	
UK	16
India	6
Religion	
Hindu	10
Muslim	1
Sikh	4
Christian	2
Atheist	3
Prefer not to say	2
Level of education	
College/pre-college	6
University degree	8
Postgraduate degree	8
Employment status	
Full-time employment	15
Part-time employment	3
Self-employed	1
Student	3
Ongoing health issues	
None	15
Hypertension	1
Type 2 diabetes	1
Asthma	3
Hypothyroidism	1
Yes, but prefer not to say	1

### Thematic analysis

Next, we sought to explore elements associated with non-participation, among minority racial and ethnic groups. Our analyses generated four overarching themes, each with subthemes that reflect the cultural, social, and practical factors influencing participation in health research. These themes highlight the generational transfer of health beliefs, barriers to and motivators for research engagement, preferences for study design and the communication approaches required to build trust.

### Theme 1. generational and cultural influences on health practices

#### Reliance on home remedies

Traditional remedies remained a strong feature of family health practices. Participants described the routine use of spices, herbal oils and homemade mixtures as a first line of care, passed down through older relatives and reinforced by everyday conversations in the community (Q1). These practices were often valued not only for their perceived effectiveness but also as expressions of cultural identity. The use of home remedies was not seen as incompatible with modern healthcare but as a parallel system. For many, this coexistence reflected a pragmatic approach, using what was available and trusted. However, younger participants highlighted that reliance on remedies was declining, particularly among peers accustomed to fast access to information online (Q2). This shift suggests that traditional practices may be less durable across future generations, particularly where digital resources replace oral traditions.

Q1*:* My grandparents always used herbs and spices for everything. My mum still makes coconut oil with turmeric for my skin, and I do the same for my kids.(25 years old, Black African)

Q2: Most of my friends would just Google their symptoms and order something online. It’s quicker than asking your parents for remedies.(29 years old, Black African*)*

#### Gendered transmission of knowledge

The role of women as custodians of traditional health knowledge was strongly emphasised. Older women, particularly mothers and grandmothers, were often the first point of contact when someone became ill (Q3). This was described as a natural extension of women’s roles as carers and household managers in previous generations. While valued, this dynamic sometimes limited younger adults’ autonomy, as advice from older relatives could discourage formal medical care or research participation (Q3). At the same time, younger women in particular felt they were inheriting this responsibility, balancing respect for tradition with their own preference for professional healthcare.

Q3: It was always the aunties who knew what to do. If someone was sick, you’d go to a neighbour or relative before you went to the doctor*.* (25 years old, Black African)

#### Shifting attitudes among younger generations

Interviews revealed clear generational differences. Participants expressed greater confidence in modern medicine and were more open to preventative care and formal research participation compared with their anecdotal experiences and perceived attitudes of their family elders. However, they also voiced ambivalence about losing cultural knowledge (Q4). This intergenerational tension reflected broader identity negotiations, between continuity of heritage and integration into mainstream systems of healthcare and research.

Q4*:* I don’t think many young people will carry it on. Everything’s online now, so unless it’s happening in your family, you won’t know. (29 years old, South Asian)

### Theme 2. experiences and barriers in health research participation

#### Limited prior engagement

Across both South Asian and Black groups, participants consistently reported little or no prior involvement in research (Q5). This absence was often interpreted as evidence that recruitment strategies did not actively include people like them. For many, the lack of outreach reinforced perceptions that research was ‘for others’, typically older adults with chronic conditions or people more connected to academic or clinical networks (Q5).

Q5: I’ve never once been asked to take part in a study. It feels like they don’t look for people like us*.* (29 years old, Black African)

#### Time pressures and competing priorities

Participants highlighted their life stage as a key barrier. Juggling work, studies, childcare and household responsibilities left little capacity to commit to additional activities (Q6). Even where interest existed, research was often perceived as low priority compared with immediate daily obligations. This practical barrier intersected with issues of accessibility; studies requiring travel or long sessions were considered unrealistic.

Q6: After work and looking after the kids, there’s no time left. Research just doesn’t fit into everyday life. (30 years old, South Asian)

#### Language barriers and mistrust

Although younger participants were generally fluent in English, they frequently described how language barriers restricted older relatives’ ability to engage with research. Misunderstanding consent forms or technical terms often generated suspicion and disengagement (Q7). Even among younger adults, trust hinged on clarity around data use. While many were comfortable sharing personal information if they understood its purpose, lack of transparency quickly led to doubts (Q8).

Q7: If you don’t understand the paperwork, you’re not going to sign it. That’s why my dad would never do it. (25 years old, South Asian)

Q8: I don’t mind giving my data if I know how it’s used. But when it’s not explained properly, that’s when people get suspicious. (27 years old, South Asian)

#### Scepticism and perceived irrelevance

Some participants, particularly when describing older relatives, emphasised scepticism towards research. This was framed as a belief that lived experience and traditional knowledge outweighed formal studies (Q9). This sense of irrelevance suggested that research must demonstrate tangible benefits for participants’ everyday lives to overcome such attitudes.

Q9: My uncle has had diabetes for decades. He says, ‘I know how to manage it, I don’t need some research to tell me what to eat. (28 years old, South Asian)

### Theme 3. motivators and preferences for research participation incentives and practical support

Young adults strongly endorsed the value of modest incentives. Vouchers and reimbursements were described as both a sign of respect for participants’ time and a practical necessity in encouraging engagement (Q10). Flexible scheduling and short, accessible study formats were also identified as essential.

Q10: If it was a quick survey with a voucher, I’d do it. It makes you feel like your time is valued. (26 years old, South Asian)

#### Comfort with non-invasive studies

There was broad consensus that non-invasive research methods, such as surveys or observational studies, were most acceptable (Q11). Invasive procedures and drug trials were consistently rejected, with safety and risk to family responsibilities cited as deterrents. Even where researchers provided assurances, participants stressed that trust could not always overcome fears of side effects (Q12).

Q11: I’d happily answer questions, but I wouldn’t risk trying new treatments. I’ve got kids depending on me. (24 years old, South Asian)

Q12: You can explain the safety over and over, but people will still be scared of side effects*.* (22 years old, South Asian)

#### Importance of peer and community influence

Word of mouth emerged as a central motivator (Q13). Hearing about positive experiences from friends or respected community members often carried more weight than formal information (Q13). This reflects the importance of relational trust and community-based dissemination in shaping willingness to engage.

Q13: If someone I knew, did it and said it was fine, I’d be much more likely to take part. Word of mouth carries weight. (24 years old, South Asian)

### Theme 4. communication and trust-Bbuilding

#### Preferred channels by age group

Participants emphasised the importance of tailoring communication strategies by generation. Younger adults leaned towards social media platforms, including Instagram, YouTube and podcasts, which they described as familiar and engaging (Q14). Older relatives, by contrast, were more responsive to interpersonal outreach, posters in community centres and announcements within religious institutions (Q14).

Q14: My mum would notice a poster in the temple, but I’d be more likely to see it on Instagram. (19 years old, South Asian)

#### Language accessibility and representation

Clear, jargon-free language was highlighted as critical. Translation into South Asian and African languages was considered essential for older adults. Beyond language, participants stressed the value of representation which underscored how visible cultural alignment could reinforce trust and participation (Q15).

Q15: If the researcher looks like me and speaks my language, it makes it feel safe. You trust them more*.* (29 years old, Black African)

#### Building trust through transparency

Participants emphasised that researchers should be open about the purpose of data collection, storage and disposal (Q16). Tangible reassurances about confidentiality and limits of use were seen as important in overcoming suspicion.

Q16: If you tell people straight that the data won’t be sold and will be deleted after a time, they’ll feel more comfortable. (27 years old, South Asian)

#### Health priorities identified by communities

When discussing priority health concerns, participants pointed to conditions disproportionately affecting their communities, including diabetes, hypertension and cardiovascular disease (Q17). Mental health was also raised, though stigma remained a major barrier to open dialogue (Q17). These priorities suggest that aligning research agendas with visible community needs could foster more meaningful engagement.

Q17: In our community, everyone talks about diabetes and blood pressure. But nobody talks about mental health, even though people are struggling*.* (28 years old, South Asian)

## Discussion

This novel qualitative study explored the perspectives of young adults from South Asian and Black communities in the UK on participation in health research. Participants described the influence of cultural traditions, generational differences, and practical constraints such as time, trust and communication. There was a clear emphasis on the preference for non-invasive research, the importance of transparency and the role of incentives and peer endorsement in shaping willingness to engage. While these findings reflect several themes previously established in the literature,^2^ the focus on younger adults herein provides new insights from a group whose voices remain under-represented in wider discussions of research inclusivity.^24^

Existing evidence has consistently shown that ethnic minority groups are less likely to be recruited into and retained within health research compared with White populations in the UK.^25^ This in part can also be seen in the UK Biobank, whereby approximately 94% of participants identify as White, with only around 6% representing all other ethnic groups combined.^26^ Furthermore, analysis of data herein from the Royal Wolverhampton NHS Trust reinforces this pattern with White participants constituting 76.5% of the total 524 participants enrolled in health research projects as of January 2025, while Asian and Black participants accounted for only 11.6% and 4.8%, respectively. Notably, no Black participants were recruited into commercial studies, indicating particularly low representation in certain research contexts. These values are in stark contrast to the local population, which is only 60.5% White but upwards of 21.1% Asian and 9.3% Black. These figures mirror national trends and underscore the persistence of ethnic disparities in research participation, even in regions where diverse communities are present.

Much of existing research on this topic in the UK has concentrated on middle-aged populations, often because they are disproportionately affected by chronic diseases such as diabetes and cardiovascular conditions.^27^ Nonetheless, several systematic reviews have highlighted existing barriers to health research for ethnic minorities in the UK including language difficulties, cultural mismatch, mistrust of institutions and lack of awareness.^28–31^ These barriers are not limited to the UK, with other western countries illustrating similar findings, including the USA, Australia, Germany and Spain,^32–34^ which only further highlights and compounds health inequalities present within global healthcare provision. Nonetheless, our data extend the conversation by illustrating that while these issues may remain equally relevant to younger adults, they likely present in alternative ways.^32^ For example, rather than language barriers which are regularly cited in older populations,^31^ younger participants often described time pressures, competing priorities and limited awareness of opportunities as the main reasons for non-participation. This highlights an important gap in the literature: the tendency to focus on older populations has meant that the experiences and perspectives of younger ethnic minority adults, who may play a crucial role in influencing family health behaviours and future research participation remain largely unexplored. Our findings are consistent with previously identified barriers but importantly we highlight the distinct pressures and opportunities faced by younger adults in an ever-evolving society. Unlike their parents and grandparents, younger people often combine cultural health practices with digital information-seeking,^35^ reflecting hybrid health identities shaped by both tradition and modernity.

We herein reinforce the emerging literature suggesting that younger adults act as ‘cultural mediators’ within families and communities. Prior research has shown that children and young adults often translate medical advice, interpret consent forms and guide older relatives in navigating healthcare systems. By engaging younger people in health research, there is potential to influence wider family participation and reshape community trust. This is particularly relevant in ethnic minority populations where health decisions are frequently collective rather than purely individual.

Our findings contribute to broader debates on equality, diversity and inclusion (EDI) in research. Over the last decade, policy frameworks in the UK have called for greater inclusivity in clinical trials and public health research, yet progress has been uneven and, in some cases, remains passive advice. For example, in 2022 the National Institute for Health and Care Research (NIHR) launched its Research Inclusion Strategy 2022–2027, committing research culture, systems and practices to more equitable participation of under-represented groups.^36^ Similarly, the NIHR Race Equality Framework emphasises that public involvement and participation should be representative of those communities most affected by health inequalities.^37^ The UK Government’s Inclusive Britain report (2024) explicitly aims to improve ethnic minority representation in clinical trials to increase trust and relevance of research to the population.^38^ More recently, the Association of Medical Research Charities and Association of the British Pharmaceutical Industry’s 2025 ‘Achieving Inclusivity in Clinical Research’ report has called for a UK-wide strategy, improved measurement of trial diversity and sharing of best practice across the sector.^39^ Despite this, recruitment strategies in clinical research often adopt a one-size-fits-all approach, prioritising convenience for research teams over accessibility for participants. The NIHR INCLUDE roadmap emphasises the necessity of embedding inclusivity at every stage of the research process, from study design to dissemination.^16^ It outlines strategic intervention points to enhance the inclusion of under-served groups, advocating for sustained investment in building trust, providing culturally relevant information and respecting participants’ time and constraints. While these initiatives and strategies are positive, they stop short of actively enforcing ethnic diversity in study recruitment.^29^ Internationally, more direct approaches have been adopted, such as the National Institute of Health’s in the USA mandate for the inclusion of participants from ethnic minority groups.^28^

### Recommendations

Our qualitative study of young adults aged 18–30 from Black and Asian backgrounds provides important insights with relevance to public health, health services research and patient-facing clinical disciplines. While grounded in a UK context, the findings have broader applicability to settings internationally where similar patterns of under-representation and mistrust in research participation are observed.

The study highlights several priorities for future research aimed at improving equity in participation. First, there is a need to rigorously evaluate targeted recruitment strategies designed to engage younger adults from under-represented ethnic groups, ensuring that these approaches are grounded in lived experiences and culturally relevant contexts. Second, future intervention studies should assess the effectiveness of combining traditional recruitment approaches with digital platforms, including social media and podcasts. Such blended strategies may have relevance across diverse health systems, particularly in settings with high digital engagement among younger populations. Third, further research should examine the role of ethically approved incentives, particularly when paired with transparent communication about data use and research purpose. This combined approach may help address mistrust in a range of international contexts while maintaining ethical integrity. Finally, longitudinal research is needed to explore whether early engagement with research during young adulthood fosters sustained participation over the life course. If effective, such approaches could contribute to shifting entrenched patterns of under-representation across generations and across health systems. Collectively, these findings offer a transferable framework to inform more inclusive recruitment strategies in health research globally, particularly in multicultural and underserved populations.

### Limitations

Our study has several limitations. Black adults were under-represented, likely reflecting the demographics of our existing recruitment networks and the communities accessed, which limited the diversity of perspectives and comparative insights across ethnic groups. Collectively, this perhaps reflects the wider challenges associated with recruitment and securing participants for research and concepts of participants wanting to connect with researchers who ‘look and sound like me’. Furthermore, women predominated the participation which may in part be due to the fact both interviewers were female. Recruitment via digital and peer networks may have excluded individuals with lower connectivity or weaker social ties, potentially biasing the sample toward those with digital access and more digitally engaged or accessible participants. Finally, the short timeframe constrained (ie, funding limitations) opportunities to follow-up emerging themes or recruit a more balanced sample, limiting the depth and breadth of the data. Despite these limitations, the study provides valuable insights into younger adults’ experiences, highlighting themes that can inform more inclusive recruitment and engagement strategies in future research.

## Conclusion

This study highlights how addressing under-representation in health research depends on understanding the intersecting cultural, generational and social factors that shape participation. By foregrounding the perspectives of younger South Asian and Black adults, it identifies a critically under-engaged group with the potential to reshape research engagement and strengthen community participation.

The findings call for a shift away from generic recruitment towards approaches that are targeted, digitally enabled and culturally resonant, underpinned by transparency and ethical practice. Embedding inclusivity as a core principle of study design has the potential to improve participation, enhance the representativeness of health data and strengthen the validity and relevance of research used to inform policy and practice. These insights have also been translated into an evidence-informed video resource to support researchers in adopting more inclusive recruitment strategies (https://tinyurl.com/4z5mudp3).

## Supplementary material

10.1136/bmjopen-2026-122025 online supplemental file 1

## Data Availability

Data are available upon reasonable request. All data relevant to the study are included in the article or uploaded as supplementary information.

## References

[1] NIHR (2024). Addressing health and care needs and priorities.

[2] Redwood S, Gill PS (2013). Under-representation of minority ethnic groups in research--call for action. Br J Gen Pract.

[3] Treweek S, Pitkethly M, Cook J (2018). Strategies to improve recruitment to randomised trials. Cochrane Database Syst Rev.

[4] Subramanian A, Adderley NJ, Gkoutos GV (2021). Ethnicity-based differences in the incident risk of allergic diseases and autoimmune disorders: A UK-based retrospective cohort study of 4.4 million participants. Clin Exp Allergy.

[5] Pham TM, Carpenter JR, Morris TP (2019). Ethnic Differences in the Prevalence of Type 2 Diabetes Diagnoses in the UK: Cross-Sectional Analysis of the Health Improvement Network Primary Care Database. Clin Epidemiol.

[6] Fernando E, Razak F, Lear SA (2015). Cardiovascular Disease in South Asian Migrants. Can J Cardiol.

[7] Kooner A, Kooner IK, Misra S (2024). 203 Cumulative incidence of coronary heart disease (CHD) among UK South Asians – results of the prospective follow-up of the London Life Sciences Population (LOLIPOP) cohort of 25,372 participants, at 20 years. BMJ.

[8] Mathur R, Rentsch CT, Morton CE (2021). Ethnic differences in SARS-CoV-2 infection and COVID-19-related hospitalisation, intensive care unit admission, and death in 17 million adults in England: an observational cohort study using the OpenSAFELY platform. The Lancet.

[9] Morales DR, Ali SN (2021). COVID-19 and disparities affecting ethnic minorities. The Lancet.

[10] Khubchandani J, England-Kennedy E, Wiblishauser M (2025). Engaging community members in health-related research and interventions. *Journal of Medicine, Surgery, and Public Health*.

[11] Holzer JK, Ellis L, Merritt MW (2014). Why we need community engagement in medical research. J Investig Med.

[12] Pan D, Sze S, Minhas JS (2020). The impact of ethnicity on clinical outcomes in COVID-19: A systematic review. EClinicalMedicine.

[13] Public Health England (2020). Disparities in the risk and outcomes of covid-19.

[14] Williams AM, Clayton HB, Singleton JA (2022). Racial and Ethnic Disparities in COVID-19 Vaccination Coverage: The Contribution of Socioeconomic and Demographic Factors. Am J Prev Med.

[15] Hussain-Gambles M, Atkin K, Leese B (2004). Why ethnic minority groups are under-represented in clinical trials: a review of the literature. Health Soc Care Community.

[16] Witham MD, Anderson E, Carroll C (2020). Developing a roadmap to improve trial delivery for under-served groups: results from a UK multi-stakeholder process. Trials.

[17] Office for National Statistics (2021). 2021 UK census.

[18] Pines RL, Jones L, Sheeran N (2020). Using Family Members as Medical Interpreters: An Explanation of Healthcare Practitioners’ Normative Practices in Pediatric and Neonatal Departments in Australia. Health Commun.

[19] Ofcom (2023). Online nation 2023 report. https://www.ofcom.org.uk/siteassets/resources/documents/research-and-data/online-research/online-nation/2023/online-nation-2023-report.pdf?v=368355.

[20] Chen J, Wang Y (2021). Social Media Use for Health Purposes: Systematic Review. J Med Internet Res.

[21] Judith G, Nicki T (2018). Qualitative Methods for Health Research.

[22] Saunders B, Sim J, Kingstone T (2018). Saturation in qualitative research: exploring its conceptualization and operationalization. Qual Quant.

[23] Bispo Júnior JP (2022). Social desirability bias in qualitative health research. Rev Saude Publica.

[24] Smart A, Harrison E (2017). The under-representation of minority ethnic groups in UK medical research. Ethn Health.

[25] Haley SJ, Southwick LE, Parikh NS (2017). Barriers and Strategies for Recruitment of Racial and Ethnic Minorities: Perspectives from Neurological Clinical Research Coordinators. J Racial Ethn Health Disparities.

[26] Fry A, Littlejohns TJ, Sudlow C (2017). Comparison of Sociodemographic and Health-Related Characteristics of UK Biobank Participants With Those of the General Population. Am J Epidemiol.

[27] Miles G, Smith RA (2024). 2051 How representative are UK-based trials investigating lifestyle interventions for diabetes mellitus: a systematic review. Age Ageing.

[28] Pardhan S, Sehmbi T, Wijewickrama R (2025). Barriers and facilitators for engaging underrepresented ethnic minority populations in healthcare research: an umbrella review. Int J Equity Health.

[29] Masood Y, Bower P, Waheed MW (2019). Synthesis of researcher reported strategies to recruit adults of ethnic minorities to clinical trials in the United Kingdom: A systematic review. Contemp Clin Trials.

[30] Bodicoat DH, Routen AC, Willis A (2021). Promoting inclusion in clinical trials-a rapid review of the literature and recommendations for action. Trials.

[31] Spencer LH, Cooledge BA, Hoare Z (2025). The experiences of minority language users in health and social care research: A systematic review. Int J Health Plann Manage.

[32] George S, Duran N, Norris K (2014). A systematic review of barriers and facilitators to minority research participation among African Americans, Latinos, Asian Americans, and Pacific Islanders. Am J Public Health.

[33] Shea L, Pesa J, Geonnotti G (2022). Improving diversity in study participation: Patient perspectives on barriers, racial differences and the role of communities. Health Expect.

[34] Ekezie W, Cassambai S, Curtis F (2026). Global Insights on the Involvement of Ethnic Minority Populations in Health and Social Care Research Priority Setting: A Systematic Scoping Review. J Racial Ethn Health Disparities.

[35] Radu I, Scheermesser M, Spiess MR (2023). Digital Health for Migrants, Ethnic and Cultural Minorities and the Role of Participatory Development: A Scoping Review. Int J Environ Res Public Health.

[36] NIHR Research inclusion strategy 2022-2027.

[37] National Institute of Health and Social Research (2025). NIHR race equality framework.

[38] Government of UK (2024). Inclusive Britain second update report.

[39] Association of Medical Research Charities (2025). Achieving inclusivity in clinical research.

