# Supplementary material for: Four decades of under-representation: a qualitative study to gain young adult insights to transform inclusive recruitment in clinical research
Source: BMJ Open. 2026 Oct 26;16(10):e122025. doi: 10.1136/bmjopen-2026-122025 (PMC13711570; doi:10.1136/bmjopen-2026-122025)
Supplement: Supplementary file 1 [file bmjopen-2026-122025.reviewer_comments.pdf]

## PEER REVIEW HISTORY

BMJ Open publishes all reviews undertaken for accepted manuscripts. Reviewers are asked to complete a checklist review form and are provided with free text boxes to elaborate on their assessment. These free text comments are reproduced below.

### ARTICLE DETAILS

**Title (Provisional)**

Four decades of underrepresentation: A qualitative study to gain young adult insights to transform inclusive recruitment in clinical research

**Authors**

Kumar, Kanta; Agarwal, Vanshika; Trivedi, Palak J; Greenfield, Sheila; Leung, Elaine; Jacques, Lorraine; Quinlan, Jonathan I

---

### VERSION 1 - REVIEW

---

| | |
|---|---|
| **Reviewer** | **1** |
| **Name** | **Cole, Helen** |
| **Affiliation** | **Universitat Autònoma de Barcelona, Pediatrics, Obstetrics, Gynecology, Preventive Medicine and Public Health** |
| **Date** | **30-Jun-2026** |
| **COI** | **None** |

---

The paper addres an important issue and makes good use of a qualitative research design. However, my main concerns are that the results are misaligned with the research questions (see Theme 1, which does not clearly relate to engagement with health research, but to health practices in general, and this departs from the intended purpose of the article). Furthermore, the authors should conduct a more thorough literature review and discuss more what the potential implications are of excluding ethnic and racial minority groups from research, perhaps drawing on historic or current clinical examples.

Abstract

It's not clear how the two types of data used in the analysis are used in the current study.

Strengths/Limitations

The first strength is not very unique.

In the second strength, what is meant by "use of public and patient involvement"?

Introduction

I think it would be important to explain better what the clinical or public health significance of the problem is. That is, explain better why we can't just extrapolate the results of a less diverse sample to benefit people of all races/ethnicities, and therefore why ethnic minorities tend not to benefit as much from the results of clinical research studies.

Methods

The quantitative methods are not described despite that the first research question relates to the quantitative data. How was the data analyzed?

The recruitment methods are a bit unclear. How did you gain access and permission to recruit (if required) from those informal networks? Did you search online for organizations, did you use your own personal contacts, or some other way to find those networks?

The development of the research guide does not clearly describe how the content was linked to the research questions. Although it's important to use the COREQ checklist, I don't believe it's a design tool, rather a checklist for reporting. Rephrase the way you describe the study design so that you explain how you address your research questions rather than how you relied on the COREQ checklist.

The data analysis section is split in to too many small subsections. It would be good to provide more narrative connections between each.

The section about Rigor needs to be rewritten so that it describes the importance of each technique and how these techniques were applied.

Results

For those not familiar with the Royal Wolverhampton NHS Trust Research and Development Department, explain what the significance and scope of the data analyzed is. Is this data from all studies conducted by this group, or only a subset, is this data representative of all those participating in certain types of research, or not?

In the section about Generational differences, what age groups are you comparing given that the participants were all under 30? Or are you reporting the perceived attitudes of older groups according to the perceptions of the young adults interviewed?

It's unclear how the information in Theme 1 relates to the research questions as stated in the introduction, if at all. Did participants link these experiences and knowledges to engagement with health research at all?

The quotes would be more effective if integrated with in the text and linked through in a more narrative way.

Discussion

Better engagement with existing literature on the topic, both current and historic is needed. There are several systematic reviews from recent years for example on barriers and

facilitators to participation in research among racial and ethnic minority groups. What does this paper provide that is unique from existing literature?

Although this article is based in the UK, perhaps relating it to some of the literature from the US context, where this topic has perhaps been more thoroughly examined, would help to contextualize the discussion better and to make a case for the importance of the topic.

---

| | |
|---|---|
| **Reviewer** | **2** |
| **Name** | **Beauchemin, Melissa** |
| **Affiliation** | **Columbia University, The Mailman School of Public Health** |
| **Date** | **02-Aug-2026** |
| **COI** | **None** |

---

Thank you for the opportunity to review this manuscript that aims 1) To quantitatively evaluate NHS trial recruitment data within the UK West Midlands; 2) To explore how young South Asian and Black adults view their own participation in health research, and whether this aligns with long-standing patterns of exclusion; 3) To understand how these young adults believe their parents, elders, and community members could be effectively engaged in research, effectively positioning them as potential ambassadors for inclusive approaches.

Overall, the authors ask an interesting question and focus on an important topic of underrepresentation on clinical trials; however there are some major concerns that raise questions.

Abstract:

The abstract seems oddly laid out with the listed strengths/limitations at the end. Consider integrating into the text.

Background:

The authors take an interesting stance and justify the importance of understand representation (and underrepresentation) especially as COVID made the gaps in healthcare disparities and access and participation more stark than ever.

Methods:

The justification for the 2 studies combined together is not clear. In a sense, this is a multiple methods study; however, this is not clearly articulated and in the end, it feels like the studies as just put together without considering methodological challenges/strengths.

Further, the authors state they used an “interpretivist paradigm” but what this means and how it is applied methodologically is not clear. For example, there are no citations or references to where/how this has been used before and why it is justified for this question.

There are major concerns about the recruitment/sampling procedures. The broad availability of potential participants seems difficult to match with the ultimate 22 interviews that were conducted.

The number of interviews should go in results.

COREQ checklist is mentioned multiple times, but the explanations are superficial. Matching these criteria to how the study was designed and implemented are needed. For example, the section on rigor reads like a checklist and does not directly connect to the study design, question, or procedures.

The methods of the retrospective analysis of enrollment are not explained. There is only a brief (2nd) paragraph in methods). How were these data considered together?

Results:

It is difficult to interpret the results and provide critique given the major concerns in the methods.

---

## VERSION 1 - AUTHOR RESPONSE

**Reviewer 1**

The paper addresses an important issue and makes good use of a qualitative research design. However, my main concerns are that the results are misaligned with the research questions (see Theme 1, which does not clearly relate to engagement with health research, but to health practices in general, and this departs from the intended purpose of the article). Furthermore, the authors should conduct a more thorough literature review and discuss more what the potential implications are of excluding ethnic and racial minority groups from research, perhaps drawing on historic or current clinical examples.

Abstract

It's not clear how the two types of data used in the analysis are used in the current study.

*Response: Thank you for this comment. We agree that throughout the manuscript, inclusive of the abstract, it was not clear how the two datasets are connected. In this case, additional text has been added into the abstract to help explain how the data was used whilst keeping the abstract brief (Line 66-68).*

Strengths/Limitations

The first strength is not very unique.

*Response: We agree, and accept this criticism This strength has now been removed.*

In the second strength, what is meant by "use of public and patient involvement"?

*Response: The wording in this section has now been amended to better represent this strength. Specifically, public partners were involved in the creation of the interview guide and agreement of findings and themes (Line 96).*

Introduction

I think it would be important to explain better what the clinical or public health significance of the problem is. That is, explain better why we can't just extrapolate the results of a less diverse sample to benefit people of all races/ethnicities, and therefore why ethnic minorities tend not to benefit as much from the results of clinical research studies.

*Response: Thank you for this comment, we have now added additional text within the second paragraph to bring this point into focus (Lines117-120)*

Methods

The quantitative methods are not described despite that the first research question relates to the quantitative data. How was the data analyzed?

*Response: We acknowledge it has not been clear how this data was analysed. Further description of the NHS data has now been moved up in the manuscript with additional details added as to how the data was analysed (Lines179-184).*

The recruitment methods are a bit unclear. How did you gain access and permission to recruit (if required) from those informal networks? Did you search online for organizations, did you use your own personal contacts, or some other way to find those networks?

*Response: Additional detail has been provided within text (Lines 197-198). However, additionally for the reviewer's clarity, these relationships have been built up over years of research, engagement within local communities and partnerships with public partners or community leaders.*

The development of the research guide does not clearly describe how the content was linked to the research questions. Although it's important to use the COREQ checklist, I don't believe it's a design tool, rather a checklist for reporting. Rephrase the way you describe the study design so that you explain how you address your research questions rather than how you relied on the COREQ checklist.

*Response: Thank you for this comment, we agree with this and as such additional detail has been provided to describe the development of the interview topic guide (Lines 218-222).*

The data analysis section is split in to too many small subsections. It would be good to provide more narrative connections between each.

*Response: We agree with this comment, and as such this section has now been reworked and several sections have been combined to leave 3 key sections. We hope this is satisfactory.*

The section about Rigor needs to be rewritten so that it describes the importance of each technique and how these techniques were applied.
*Response: Thank you for this comment. This section has now been rewritten in a descriptive format rather than the previous list, providing details of the importance of each component as well as how these were applied herein.*

Results
For those not familiar with the Royal Wolverhampton NHS Trust Research and Development Department, explain what the significance and scope of the data analyzed is. Is this data from all studies conducted by this group, or only a subset, is this data representative of all those participating in certain types of research, or not?

*Response: Some further clarity has been added to ensure it is clear these data come from enrolled participants within the NHS trust as of January 2025. The studies themselves were not conducted, or led by any of the author team.*

In the section about, what age groups are you comparing given that the participants were all under 30? Or are you reporting the perceived attitudes of older groups according to the perceptions of the young adults interviewed?

*Response: Thank you for the comment. We agree this can appear confusing as the study only recruited younger adults. To confirm, there was no direct group comparisons, but instead this is reporting the perceived attitudes according to the younger adults and reporting of behaviours, opinions, and experiences of their elder family members. Some wording has been added on lines 387-388 to reflect this, and we hope this provides further clarity.*

It's unclear how the information in Theme 1 relates to the research questions as stated in the introduction, if at all. Did participants link these experiences and knowledges to engagement with health research at all?

*Response: We acknowledge that theme 1 is perhaps more related to general health practices and/or beliefs with preference of home remedies and traditional medicines as*

*opposed to a direct focus on their engagement in health research. However, to understand why people may or may not engage in health research, we felt it important to understand their general perception of health practices and their health decisions. In turn, these data may in part explain a reluctance in older generations to engage in health research aimed at advancing modern medicines due to their traditional preferences, cultural influences and gender roles. In contrast, it was clear the younger adults would seek modern medicines and knowledge via internet sources as opposed to family remedies. As such, we have added a fourth aim within the introduction for full clarity.*

The quotes would be more effective if integrated with in the text and linked through in a more narrative way.

*Response: Thank you for the comment. The quotes have now been integrated within the text and linked in this way. As a consequence, the original table 2 has now been removed.*

Discussion
Better engagement with existing literature on the topic, both current and historic is needed. There are several systematic reviews from recent years for example on barriers and facilitators to participation in research among racial and ethnic minority groups. What does this paper provide that is unique from existing literature?

*Response: We acknowledge the reviewer's point and agree that further discussion would be helpful to provide wider context. As such, further discussion has been added with additional references to address this. In addition, we have reworded some sections to further highlight how our paper provides unique perspectives compared with existing literature (Lines 541-545).*

Although this article is based in the UK, perhaps relating it to some of the literature from the US context, where this topic has perhaps been more thoroughly examined, would help to contextualize the discussion better and to make a case for the importance of the topic.

*Response: Thank you for this comment. Some additional discussion has been added to highlight that these issues are not strictly UK only, and in fact a global issue (Lines 541-545).*

**Reviewer: 2**
Thank you for the opportunity to review this manuscript that aims 1) To quantitatively evaluate NHS trial recruitment data within the UK West Midlands; 2) To explore how young South Asian and Black adults view their own participation in health research, and

whether this aligns with long-standing patterns of exclusion; 3) To understand how these young adults believe their parents, elders, and community members could be effectively engaged in research, effectively positioning them as potential ambassadors for inclusive approaches.

Overall, the authors ask an interesting question and focus on an important topic of underrepresentation on clinical trials; however there are some major concerns that raise questions.

Abstract:
The abstract seems oddly laid out with the listed strengths/limitations at the end. Consider integrating into the text.

*Response: Thank you for this comment, and we do agree that this may seem strange added to the end of the abstract. However, the strength and limitations section is a requirement of the journal and sits separately from the abstract itself. We believe this is added into a standalone box within the manuscript following typesetting. In which case, please do not consider this a formal part of the abstract.*

Background:
The authors take an interesting stance and justify the importance of understand representation (and underrepresentation) especially as COVID made the gaps in healthcare disparities and access and participation more stark than ever.

Methods:
The justification for the 2 studies combined together is not clear. In a sense, this is a multiple methods study; however, this is not clearly articulated and in the end, it feels like the studies as just put together without considering methodological challenges/strengths.

*Response: Thank you for this comment and we do agree this project applies a mixed-methods approach. For clarity this was a single study that was completed (i.e., the interviews) and not two. The addition of the local NHS trust data was to provide clear regional context as to existing recruitment disparities, rather than simply relying on previously published data that may not have applied to the UK's West Midlands population, which is typically more diverse than the rest of the UK. The addition of these data further demonstrates that even in a superdiverse location, diverse recruitment to health-related studies remains limited.*

Further, the authors state they used an "interpretivist paradigm" but what this means and how it is applied methodologically is not clear. For example, there are no citations

or references to where/how this has been used before and why it is justified for this question.

*Response: Thank you for this helpful comment. We agree that the original manuscript did not sufficiently explain how the interpretivist paradigm informed the methodological approach. We have revised the Methods to define the interpretivist position, explain why it was appropriate to our research question and clarify how it informed the use of semi-structured interviews, inductive analysis and researcher reflexivity. We have also added supporting methodological references.*

There are major concerns about the recruitment/sampling procedures. The broad availability of potential participants seems difficult to match with the ultimate 22 interviews that were conducted.

*Response: Thank you for raising this concern. Following an initial advertisement of the study, a total of 33 prospective participants contacted the research team and were followed up. Consequently, 22 participants were booked in to complete the interviews. The two interviewers independently noted saturation following completion of their allotted interviews and as such a second round of recruitment was not conducted.*

The number of interviews should go in results.
*Response: This detail is now provided on lines 335*

COREQ checklist is mentioned multiple times, but the explanations are superficial. Matching these criteria to how the study was designed and implemented are needed. For example, the section on rigor reads like a checklist and does not directly connect to the study design, question, or procedures.

*Response: Thank you for this comment, and we do agree that the descriptions of how we utilised the COREQ checklist was superficial and did read like a list of items rather than providing appropriate details. As such, this section has now been rewritten in a descriptive format, providing details of the importance of each components as well as how these were applied herein.*

The methods of the retrospective analysis of enrollment are not explained. There is only a brief (2nd) paragraph in methods). How were these data considered together?

*Response: Thank you for this comment. The description of the NHS data has now been moved up in the manuscript with additional details added as to how the data was analysed.*

Results:
It is difficult to interpret the results and provide critique given the major concerns in the methods.

*Response: We appreciate the reviewer's concerns have prevented them from critiquing the results and interpretation. If appropriate, we would welcome the opportunity to receive the reviewer's comments on these sections in the next round of review and make appropriate amendments should they be required.*
