# Supplementary material for: Four decades of under-representation: a qualitative study to gain young adult insights to transform inclusive recruitment in clinical research
Source: BMJ Open. 2026 Oct 26;16(10):e122025. doi: 10.1136/bmjopen-2026-122025 (PMC13711570; doi:10.1136/bmjopen-2026-122025)
Supplement: Supplementary file 2 [file bmjopen-16-10-s001.docx]

# Harnessing Youthful Insights: Revolutionising Research Collaboration in Ethnic Minority Communities (HIGHLIGHT)

**Interview Guide for the Highlight Project**

**Project Aim:**

To understand the views of young Black and Asian adults (aged 18-30) to identify barriers and motivators for participating in health research. The project will co-design actionable recommendations to improve how health research connects with and engages diverse communities.

**Introductory Statement:**

Thank you for joining this interview. We aim to better understand the perspectives of young Black and Asian adults regarding participation in health research. Your input is invaluable for shaping inclusive strategies.

Before we start, I want to confirm that you’ve reviewed the participant information sheet and signed the consent form. This interview is entirely voluntary, and your responses will remain confidential. Do you have any questions before we begin?

**1. Cultural and Family Background**

- In your opinion, how do the traditions or values in your culture influence the community’s approach to making health-related decisions?
  *“We’re interested in how culture plays a role in everyday health choices.”*
- Just for the recordings sake, are you from XXXX background.
- Have you or anyone in your family had any experiences with health research, even if they were brief or indirect?
  *“This question focuses on any direct or indirect encounters with health research and how they might affect your attitude towards it.”*

**2. Barriers to Health Research Participation**

- From your perspective, what are the main challenges preventing people in your community from engaging with health research? And what would work for the younger / older people in your culture.
- *“We want to understand what specific factors discourage participation, such as trust, knowledge, or logistical barriers.”*
- Do you think there might be any common misunderstandings in your community about health research that might make people reluctant to participate?
  *“This will help us explore how perceptions or misunderstandings can impact participation.”*

**3. Motivators and Relevance**

- What would make you feel more confident or excited about taking part in health research?
  *“We’re curious about what would encourage you or others to actively get involved in health research.”*
- (Do you think that addressing health topics specifically relevant to your community would make research more engaging?) – Only ask if participant responds to the above
  *“We want to know whether focusing on community-specific health issues could increase participation and why.”*

**4. Recommendations and Solutions**

- Considering what you’ve told me, what types of studies do you think we need to pay attention to, when asking people to take part? Let me give you some examples, perhaps we’re testing a medicine, we’re doing something observational and just collecting data from clinic, or it might be we’re collecting a blood sample or a tissue sample, what about interviews like today?
  *“We’re looking for ideas to make research more welcoming and how community representation could strengthen it.”*
- In your view then, what’s the best way to communicate health research opportunities to your community? And again, do you think this would differ between age groups?
  *“Which methods or channels would be most effective for engaging your community?”*

**Closing Statement:**

Thank you for your insights. Your contributions will help us improve how health research connects with and supports communities like yours. Would you like to stay updated on the progress of this project, or participate in any future steps?
